# Incidence and missed diagnosis risk of occult posterior malleolar fractures associated with the tibial shaft fractures: a systematic review

**DOI:** 10.1186/s13018-021-02502-6

**Published:** 2021-06-01

**Authors:** Zhongzheng Wang, Wei Chen, Yanbin Zhu, Siyu Tian, Kuo Zhao, Jialiang Guo, Zhiyong Hou, Qi Zhang, Yingze Zhang

**Affiliations:** 1 Department of Orthopaedic Surgery, The Third Hospital of Hebei Medical University, No. 139 Ziqiang Road, Qiaoxi District, Shijiazhuang, 050051 Hebei Province People’s Republic of China; 2 Key Laboratory of Biomechanics of Hebei Province, Shijiazhuang, 050051 People’s Republic of China; 3 NHC Key Laboratory of Intelligent Orthopaedic Equipment, Shijiazhuang, 050051 People’s Republic of China

**Keywords:** Posterior malleolar fractures, Tibial shaft fractures, Diagnosis, Surgical management

## Abstract

**Background:**

Tibial shaft fractures (TSFs) combined with occult posterior malleolar fractures (PMFs) are becoming widely recognized in the field of orthopedics. The purpose of this study was to determine the clinical incidence, missed diagnosis rate, and treatment strategies of this combined injury.

**Methods:**

PubMed, Cochrane, and MEDLINE Ovid databases were searched for articles of English language from 1988 to 2020, identifying 1549 papers.

**Results:**

Twenty-one of the 1278 identified studies were eligible for inclusion. Each study reported on the incidence of this combined injury, and 12 studies documented the missed diagnosis rate. Seventeen studies reported surgical intervention strategies for PMFs. In the present review, PMFs frequently occurred in spiral TSFs (70%), especially distal third spiral TSFs (70.4%), based on CT scans or additional MRI. Based on the original X-ray detection, approximately 50% of PMFs were missed in patients with a combined injury. In addition, the treatment strategies for PMFs were inconsistent. Most studies (11/17) believe that specific surgical management needs to be developed based on the fragment size, displacement, and stability of the PMF.

**Conclusions:**

For patients with TSFs, spiral TSFs, especially distal third spiral TSFs, are closely related to PMFs and are often not sufficiently diagnosed by X-ray alone. Advanced CT and MRI examinations can significantly reduce the missed diagnosis rate of occult PMFs. According to available literature, the treatment strategy for PMFs associated with TSFs is questionable without convincing evidence of benefit.

**Supplementary Information:**

The online version contains supplementary material available at 10.1186/s13018-021-02502-6.

## Background

**Fig. 1:**
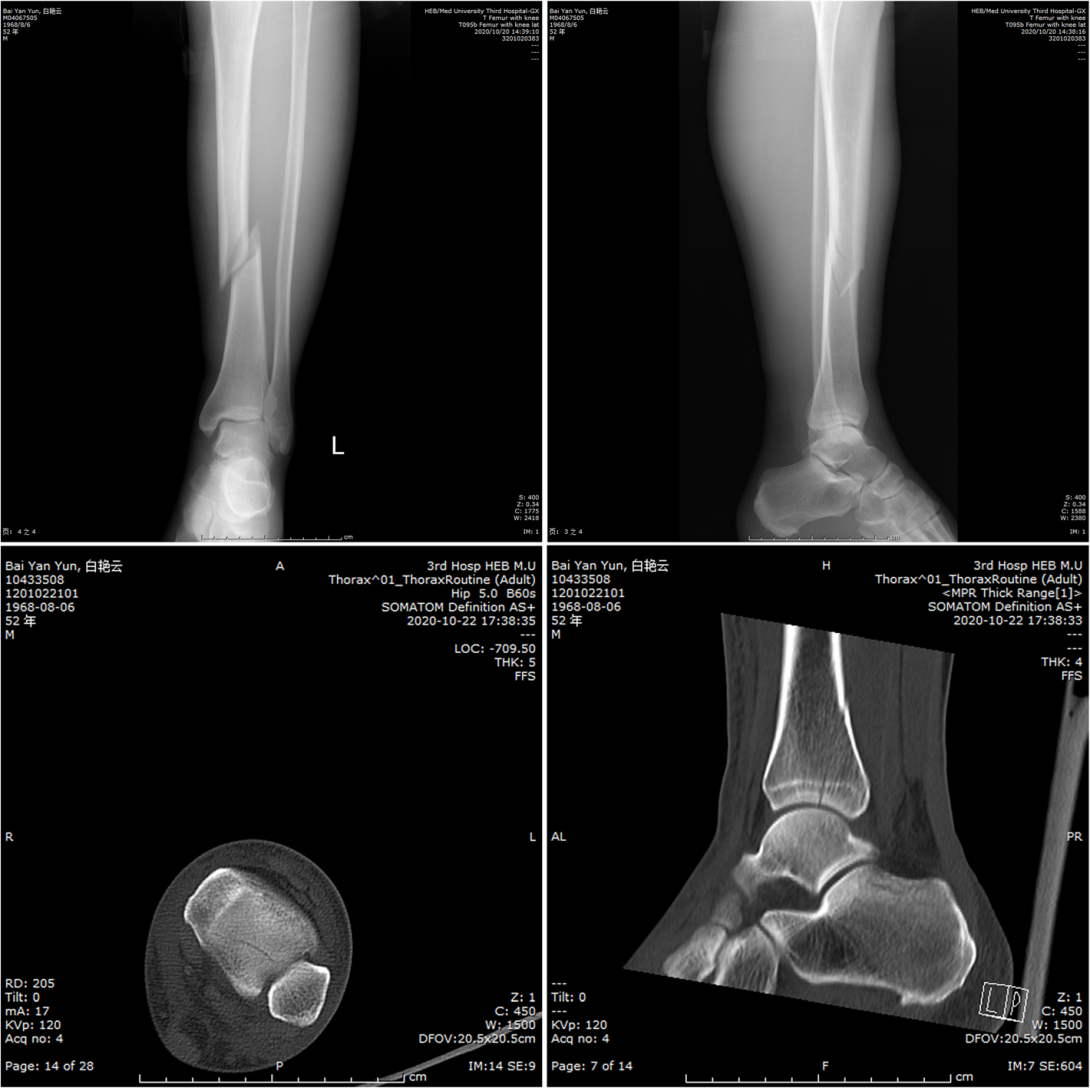
These radiological images show a non-displaced PMF associated with a TSF. **a** Anteroposterior and lateral tibia radiographs (including the ankle joint). **b** CT scans of the ankle joint (horizontal and sagittal views). The red arrow indicates the line of the PMF

Tibial shaft fractures (TSFs), especially spiral TSFs or distal third spiral TSFs, are often associated with posterior malleolar fractures (PMFs) and have been reported in several series [1–9]. However, non-displaced PMFs may be overlooked or underdiagnosed during the diagnosis and treatment of obvious TSFs (Fig. 1), which may lead to iatrogenic displacement during surgery and permanent damage to the surface of the ankle [3, 10, 11]. In the past 10 years, the published literature utilizing CT and additional MRI examinations when necessary has shown that the incidence of PMFs associated with spiral TSFs varies from 35.7 to 92.3%, which is higher than that described in previous studies [2, 8].

The reliability of radiographic assessment of PMFs has been questioned [12]. In 1988, Böstman [1] described the radiographic characteristics of TSFs combined with PMFs in detail and reported the incidence (0.9%) of this pattern of injury. Later, Boraiah et al. [13] recommended additional CT scanning of the ankle for patients with distal TSFs to prevent missed diagnoses in 2008. Subsequently, Hou et al. [2] found that tibial diaphyseal spiral fractures were commonly associated with occult PMFs in 2009, and the incidence of PMFs was as high as 88.2% utilizing CT and MRI detection. In addition, they also found that additional CT scanning and MRI examination could significantly reduce the rate of missed diagnoses of occult PMFs. Since then, more attention has been paid to this special combination injury in clinical practice. Sobol et al. [7] reported that the incidence of PMFs associated with distal third spiral TSFs may be even higher (92.3%) with the use of advanced imaging technology in 2018.

However, current literature shows that the PMFs associated with TSFs are often overlooked because PMFs are mostly occult and non-displaced [9, 14]. Most of the fracture lines are coronal, which are usually difficult to detect on anteroposterior or lateral plain radiographs [5, 6]. The need for fixation of the PMFs is still subject to debate. These factors undoubtedly increase the difficulty of diagnosing and treating with such complicated injuries. The objective of this review was to determine the clinical incidence, missed diagnosis rate, and treatment strategies of this combined injury.

## Methods

This systematic review of the literature was performed according to the Preferred Reporting Items for Systematic Reviews and Meta-Analyses (PRISMA) guidelines [15] (Additional file 1: PRISMA-2020-Checklist).

### Search strategy

On 18 July 2020, we searched the PubMed, Cochrane Library, and MEDLINE Ovid databases for all journal articles and conference abstracts pertaining to this topic to identify studies reporting the incidence of PMFs in patients with TSFs. Within the databases, we used the following keywords in our search: “tibial fracture” OR “tibial fractures” OR “tibial shaft fracture” OR “tibial shaft fractures” OR “spiral fracture of the tibia” OR “spiral fractures of the tibia” OR “spiral tibial fracture” OR “spiral tibial fractures” AND “posterior malleolar fracture” OR “posterior malleolar fractures.” Our search was limited to studies published between January 1988 and July 2020. We used the Boolean operator “or/and” between each search term. In addition, the lists of references of retrieved publications were manually searched for missing records. No meta-analysis was performed due to disparity of study populations, interventions, and outcome measures between the included articles.

### Study selection

Two reviewers independently screened the titles, abstracts, and full-text articles of the retrieved studies, based on the eligibility criteria. The inclusion criteria for the selection of studies were as follows: population— patients with PMFs associated with the TSFs; study design—randomized controlled trials (RCTs) and prospective and retrospective observational designs; outcome measurements—incidence, missed diagnosis rate, and treatment strategy for the combined injury; evidence—all levels of evidence; and English-language articles. The exclusion criteria for the selection of studies were as follows: pediatric patients; clinical incidence was not provided; only descriptions of surgical techniques and treatment strategies; duplicate papers; full article unavailable; and articles published in the form of a letter, comment, editorial, abstract from a scientific meeting, or case report. Any discrepancies between the two reviewers were resolved by consensus or discussion with a third reviewer [16].

### Data extraction and synthesis

The relevant study data were extracted by two independent reviewers from the final pool of included articles. The data extracted from eligible studies included the following: first author name, year of publication, study design, morphology and level of TSFs, sample size, sex, mean age, detection method, incidence of associated PMFs, energy of injury, rate of missed diagnosis of PMFs based on plain radiographs, and treatment strategy for PMFs. The main outcome measures of interest included the prevalence and missed diagnosis rate of PMFs associated with the TSFs or spiral TSFs and the treatment strategy for PMFs.

### Statistical analysis

The κ coefficient was used to assess the interrater agreement for initial and full-text screening. A κ value of ≥ 0.81 was interpreted as excellent interrater agreement, κ of 0.61–0.80 substantial agreement, κ of 0.21–0.60 fair to moderate agreement, and κ < 0.21 slight agreement [17]. Descriptive statistics were used to report the characteristics of all eligible studies, including total number of patients, mean age, energy of injury, detection methods, the incidence and missed diagnosis rate of PMFs associated with TSFs and surgical intervention strategies. The primary outcome was presented as a weighted mean, using inverse variance. All statistical analyses were performed using RStudio (RStudio, Inc., Boston, MA).

## Results

### Study identification

**Fig. 2:**
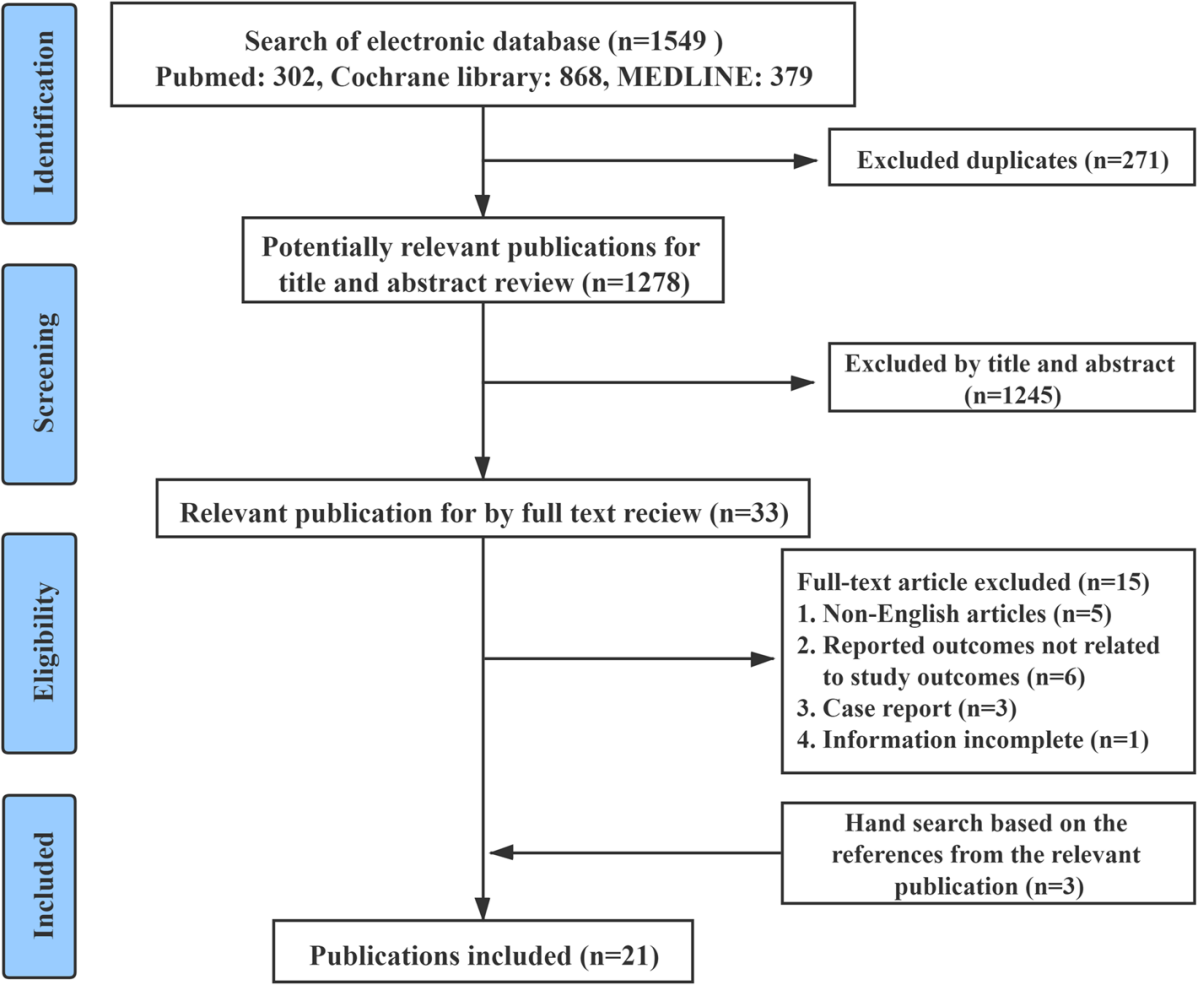
Flowchart of the systematic review protocol: study selection process

After removing duplicates from a total of 1549 related studies retrieved from the three databases, 1278 citations were screened. Thirty-three studies remained after screening the titles and abstracts. Then, a full-text review was conducted by the two reviewers. Following the review of these 33 papers, fifteen studies were excluded because they did not meet the inclusion criteria. In addition, 3 related studies were found through a manual search of the lists of references of the retrieved publications. Therefore, a total of 21 studies met our inclusion criteria and were analyzed in this systematic review, including 4 prospective studies and 17 retrospective studies. The study selection process is illustrated in Fig. 2. There was excellent agreement between reviewers at initial screening and full-text review, with κ value of 0.83 and 1.0, respectively.

### Quality and bias assessment

The details of each included study conforming to the criteria are provided in Tables 1, 2 and 3. The studies were published between 1988 and 2020. Seventeen studies were retrospective studies, and 4 of them were prospective studies [2, 4, 13, 19]. Most of the studies were judged to be of moderate to high quality according to the methodological quality tool proposed by Murad et al. [27] (see additional file 2: Quality Assessment). Inadequate description of inclusion and exclusion criteria or length of follow-up was the main cause of low quality scores. All 21 studies were assessed for risk of bias. After initial assessment, 119 of the 126 items were given the same score by both reviewers. None of the studies has an overall low risk of bias (see additional file 3: Bias Analysis Chart). Each study reported on the incidence of this combined injury, and 12 studies documented the missed diagnosis rate. Seventeen studies reported surgical intervention strategies for PMFs.

**Table 1:** Characteristics of PMFs associated with the TSFs included in the systematic review

Author, year	Study design	Tibial shaft fractures, N	Associated with PMF, N (%)	Detection method	Men, %	Mean age	Energy of injury	Rate of missed diagnosis of PMF (X-ray)	Geographic location of study	Surgical intervention
Böstman [1] (1988)	R	527	5 (0.9%)	X-ray	40%	38 (22–47)	LE	NR	Finland	Surgical fixation when recognized
Georgiadis et al. [11] (1996)	R	179	4 (2.2%)	X-ray	25%	41 (25–53)	LE	NR	USA	Surgical fixation when recognized
Kukkonen et al. [18] (2006)	R	74	18 (24.3%)	X-ray	NR	NR	94%LE	44.4%	Finland	33% underwent surgical fixation
Stuermer et al. [19] (2008)	P	214	8 (3.7%)	X-ray	NR	NR	NR	NR	Germany	Surgical fixation only when displaced
Schottel et al. [14] (2014)	R	71	18 (25.4%)	X-ray+CT	NR	NR	NR	NR	USA	Surgical fixation when large articular fragments or instability
Tsai et al. [20] (2014)	R	240	20 (8.3%)	X-ray	75%	42 (20–61)	LE	NR	China	Surgical fixation only when displaced
Jung et al. [21] (2015)	R	71	34 (47.9%)	X-ray+CT	NR	NR	NR	NR	Korea	Surgical fixation when displaced or involved over 25% of the articular surface
Kempegowda et al. [22] (2016)	R	1113	96 (9%)	X-ray+CT + MRI	63.50%	40 (18–66)	69%LE	NR	USA	73% underwent surgical fixation
			51 (4.6%)	X-ray	NR	NR	NR	46.9%		NR
			88 (7.9%)	X-ray+CT	NR	NR	NR	NR		NR
Zhang et al. [23] (2018)	R	765	55 (7.2%)	X-ray+CT	60%	45 (24–81)	NR	NR	China	NR
Huang et al. [6] (2018)	R	111	42 (37.8%)	X-ray+CT	NR	NR	NR	NR	China	Surgical fixation when displaced or involved over 25% of the articular surface
Hendrickx et al. [24] (2020)	R	263	75 (29%)	X-ray+CT	NR	NR	NR	NR	Australia	NR
Hendrickx et al. [8] (2019)	R	164	36 (22%)	X-ray+CT	75%	NR	78%LE	25%	Australia	NR

*Abbreviations*: *TSF* tibial shaft fractures, *PMF* posterior malleolus fracture; R, retrospective; P, prospective; N, numbers; NR, not reported; LE, low energy

**Table 2:** Characteristics of PMFs associated with the spiral TSFs included in the systematic review

Author, year	Study design	Tibial fractures, N	Associated with PMF, N (%)	Detection method	Men, %	Mean age	Energy of injury	Rate of missed diagnosis of PMF (X-ray)	Geographic location of study	Surgical intervention
**Spiral TSFs**										
Böstman [1] (1988)	R	129	5 (3.9%)	X-ray	40%	38 (22–47)	LE	NR	Finland	Surgical fixation when recognized
Hou et al. [2] (2009)	R	288	28 (9.7%)	X-ray	NR	NR	NR	67.9%	China	52.6% underwent surgical fixation
	P	34	3 (8.8%)	X-ray	NR	NR	NR	90%		86.7% underwent surgical fixation
			26 (76.5%)	X-ray+CT	NR	NR	NR	NR		
			30 (88.2%)	X-ray+CT + MRI	NR	NR	NR	NR		
Huang et al. [6] (2018)	R	44	29 (65.9%)	X-ray+CT	NR	NR	NR	NR	China	48.3% underwent surgical fixation
Hendrickx et al. [8] (2019)	R	48	27 (56%)	X-ray+CT	NR	NR	NR	NR	Australia	NR
**Distal third spiral TSFs**										
Purnell et al. [3] (2011)	R	27	22 (81.5%)	X-ray+CT	72.80%	48 (18–68)	60%LE	40%	USA	50% underwent surgical fixation
Warner et al. [4] (2014)	P	25	7 (28%)	X-ray	NR	NR	NR	66.7%	USA	Surgical fixation when recognized
			14 (56%)	X-ray+CT				NR		
			21 (84%)	X-ray+CT + MRI				NR		
Chen et al. [5] (2018)	R	28	10 (35.7%)	X-ray+CT	70%	51 (23–75)	LE	70%	China	Surgical fixation when recognized
Sobol et al. [7] (2018)	R	26	24 (92.3%)	X-ray+CT	NR	NR	almost LE	50%	USA	95.8% underwent surgical fixation
Hendrickx et al. [8] (2019)	R	46	27 (58.7%)	X-ray+CT	NR	NR	NR	NR	Australia	NR
**Mid-distal spiral TSF**										
Mitchell et al. [9] (2019)	R	122	59 (48.8%)	X-ray+CT	NR	NR	NR	39%	USA	51% underwent surgical fixation

*Abbreviations*: TSF, tibial shaft fractures; PMF, posterior malleolus fracture; R, retrospective; P, prospective; N, numbers; NR, not reported; LE, low energy

**Table 3:** Characteristics of PMFs associated with the distal third TSFs included in the systematic review

Author, year	Study design	Tibial fractures, N	Associated with PMF, N (%)	Detection method	Men, %	Mean age	Energy of injury	Rate of missed diagnosis of PMF (X-ray)	Geographic location of study	Surgical intervention
**Distal third TSFs (include spiral type)**										
van der Werken et al. [25] (1988)	R	148	17 (11.5%)	X-ray	58.8%	37 (27–51)	LE	47.1%	Netherlands	Surgical fixation only when displaced
Boraiah et al. [13] (2008)	R	39	13 (33.3%)	X-ray	NR	NR	NR	15.4%	USA	Surgical fixation when recognized
	P	23	11 (47.8%)	X-ray+CT				NR		Surgical fixation when recognized
Purnell et al. [3] (2011)	R	67	23 (34.3%)	X-ray. + CT	73.90%	48 (18–68)	70%LE	34.8%	USA	56.5% underwent surgical fixation
Boutin et al. [26] (2017)	R	217	42 (19.4)	X-ray+CT	NR	NR	NR	62%	USA	NR
Hendrickx et al. [8] (2019)	R	106	34 (32.1%)	X-ray+CT	NR	NR	NR	NR	Australia	NR
**Distal third TSFs (except spiral type)**										
Sobol et al. [7] (2018)	R	**167**	6 (3.6%)	X-ray	NR	NR	NR	NR	USA	NR

*Abbreviations*: TSF, tibial shaft fractures; PMF, posterior malleolus fracture; R, retrospective; P, prospective; N, numbers; NR, not reported; LE, low energy

### Patient demographics

The number of reported TSFs per study ranged from 25 to 1113. The mean age of the patients with the combined injury ranged from 37 to 51 years old. Men outnumbered women in these studies. Almost all combination fractures occurred via low-energy injuries. A total of 5021 TSFs were included in our study, 817 of which were described as spiral TSFs at different levels, 767 of which were described as distal third TSFs, and the remainder of which were only described as TSFs.

### Clinical outcomes of included studies

Each study reported on the incidence of this combined injury. For patients with TSFs, the mean incidence of PMFs was 7.3% (0.9–24.3%) based on radiographic findings [1, 11, 18–20, 22]. Based on a CT scan or MRI, the mean incidence was 25.5% (7.2–47.9%) (Table 1) [6, 8, 14, 21–24]. For patients with spiral TSFs and distal third spiral TSFs, the mean incidences of PMFs were 7.5% and 28%, respectively, based on radiographs [1, 2, 4]. Based on a CT scan or MRI, the mean incidence was 70% (56–88.2%) and 70.4% (35.7–92.3%), respectively [2–8]. Only one study provided the incidence of PMFs in patients with mid-distal spiral TSFs, which was 48.8% based on CT scans (Table 2) [9]. For patients with distal third TSFs, the mean incidence of PMFs was 28.2% based on radiographs and 33.4% based on CT scans [3, 8, 13, 25, 26]. However, excluding the spiral type of distal third TSFs, the incidence was only 3.6% based on radiographs (Table 3) [7].

Only twelve studies documented or described the missed diagnosis rate of this combined injury based on plain radiographs, which was a mean of 49.9% (15.4–90%). The gold standard of reference is a CT scan, an additional MRI examination, or discovery throughout the treatment. Seventeen studies reported surgical intervention strategies for PMFs. Among them, 6 studies indicated that surgical fixation should be performed when PMF is recognized [1, 2, 4, 5, 11, 13]; 6 studies suggested that surgical fixation should be performed when PMF is displaced, involving over 25% of the articular surface, instability, or a large fragment [6, 14, 19–21, 25]; and the remaining 5 studies only reported the percentage of surgical fixation. In patients with the combined injury who met the inclusion criteria, the treatment strategies for PMFs were inconsistent. Most of the studies (11/17) conclude that a specific surgical management is needed to develop based on the fragment size, displacement, and stability of the PMF.

## Discussion

This study is the first review was performed to comprehensively investigate the incidence, rate of missed diagnoses, and common treatment strategies for this combined injury. We found that there is a high incidence (70%, based on CT or additional MRI) of PMFs associated with spiral TSFs and distal third spiral TSFs. About half of the PMFs in this combined injury are occult and easily overlooked on plain radiographs. In these patterns of combined injuries, men are more likely to be susceptible than women, and these injuries mainly occur in the third to fourth decade, which may be related to more risky activities and are caused by low-energy injuries, including sprains, bicycle or motorcycle falls, and ski injuries. In addition, the optimal strategy for treatment of the PMF in these cases remains inconsistent.

In recent years, TSFs combined with ankle fractures have attracted increasing attention in clinical practice [9, 28]. Indeed, as early as 1946, Lauge-Hansen [29] first recognized the combination of TSFs and additional posterior malleolar injuries. Subsequently, some authors gradually observed that spiral TSFs predominantly co-occur with PMFs. The study of Böstman revealed that 5 cases (3.9%) of PMFs were associated with spiral TSFs by a preoperative X-ray [1]. However, Hou et al. [2] conducted the study in 2009 and stated that 30 cases (88.2%) of PMF were related to spiral TSFs by a CT scan or an additional MRI and found a regular “connection line” in concomitant TSF and ipsilateral PMF subsequently, which sparked research interest among orthopedic surgeons [2, 30]. Obviously, the incidence of this combined injury diagnosed by plain radiographs alone may be far underestimated. In the following 10 years, many scholars conducted the same studies and obtained similar research results. For instance, Purnell and Sobol et al. [3, 7] conducted two retrospective studies and showed that the incidence of PMFs in patients with the spiral TSFs who underwent CT scans of ankle joints was 81.5% and 92.3%. In addition, Warner et al. [4] have conducted a prospective study using the similar method as Hou [2] and found that the incidence was 84%. Therefore, it is an indisputable fact that most spiral TSFs or distal third spiral TSFs are accompanied by the PMFs.

In the present review, we have confirmed that the highest incidence of PMFs in this combined injury was mainly focused on spiral TSFs, especially distal third spiral TSFs, which may be due to the injury mechanism and special anatomical structure [5, 31–33]. Based on a CT scan or MRI, the mean incidence of PMFs in patients with spiral TSFs and distal third spiral TSFs was 70% (56–88.2%) and 70.4% (35.7–92.3%), respectively. Therefore, we believe there is strong evidence that spiral TSFs or distal third spiral TSFs have a high association with PMFs. In addition, Tables 1 and 3 shows that TSFs, distal third TSFs, or other type TSFs have a weaker correlation with PMFs.

Radiography and CT scans are commonly used to diagnose fractures; if necessary, MRI can be performed. However, the PMFs of this combined injury mostly manifest as non-displaced and crack fractures, which are challenging to diagnose only on X-ray films [2, 6, 11]. In the previous literature, few authors have systematically reviewed the missed diagnosis rate of PMFs in different injury patterns of TSFs. In the present systematic review, the mean rate of missed diagnoses was found to be 49.9% (15.4–90%) in the previous 12 studies based on X-rays. In other words, one in two patients with this combined injury had a missed diagnosis through the original radiography technology. According to our data analysis, although a routine preoperative X-ray could effectively diagnose TSFs, the diagnosis of PMFs was obviously insufficient. If not clearly diagnosed before the operation, the PMF might cause secondary displacement during or after surgery, which might result in posttraumatic arthritis of the ankle [11, 34–36]. Hence, the incidence of the combined injury was far underestimated. CT scans and an additional MRI, if necessary, of the ankle joint could significantly improve the diagnostic ability and reduce the missed diagnosis rate.

The jury is still out on the optimal treatment strategy of PMFs associated with TSFs. Generally, intramedullary nails and hollow screws or cortical lag screws are direct and effective methods to treat TSFs and PMFs [11, 37–39]. However, whether PMFs need to be fixed and the sequence of fixation—whether the TSFs or PMFs should be fixed first—have inadequate data [5, 22, 40]. A survey of American surgeons showed that there is no definite consensus on the range and standards of PMF treatment [33, 41]. Some previous studies have demonstrated that surgical intervention is recommended for all diagnosed PMFs, considering the possibility of a secondary displacement and early postoperative activity factors for PMFs [11, 22, 25, 42, 43]. However, other scholars believe that a surgical intervention is only needed for PMFs with displacement of fracture fragments ≥ 2 mm and instability or the proportion of fracture lines involving ≥ 25–30% of the articular surface [33, 34, 44–46]. When the fragments of PMFs were not displaced and small enough and if the intramedullary nail was properly inserted, the necessity of additional surgical fixation of the fragment could be eliminated [23, 40]. Of note, in a prospective study, Hou et al. [2] stated that spiral TSFs are commonly associated with PMFs, classified such combined injuries into four types, and recommended corresponding treatment strategies according to the different types of posterior malleolar injuries and the different radiologic examinations, which has a certain scientific basis. In the present review, most studies (11/17) believe that specific surgical management needs to be developed based on the fragment size, displacement, and stability of the PMF. However, no detailed study to data has been reported on the long-term clinical consequences of the PMFs associated with the TSFs. Hence, the data from the original articles did not allow us to effectively evaluate the optimal treatment for PMFs.

Some limitations of this systematic review must be noted. First, only 4 of the 21 studies included were prospectively designed. Retrospective research is susceptible to various inducement factors, leading to data bias. Second, we manually calculated some of the incidence and missed diagnosis rates in the included studies based on the data provided in the original articles. Lastly, our retrieve was limited to English articles included in the PubMed, Cochrane library, and Ovid MEDLINE databases. Therefore, not all relevant researches were included in this study.

## Conclusions

This systematic review demonstrates that the incidence and missed diagnosis rate of TSFs combined with PMFs are gradually attracting the attention of orthopedic surgeons, but the optimal strategy for treatment of the PMF in these cases is still a matter of debate.

## Supplementary Information

**Additional file 1.** PRISMA 2020 Checklist.

**Additional file 2.** Methodological Quality Assessment.

**Additional file 3.** Results of risk of bias assessment of the individual studies with scores per item.

## Data Availability

All data used and analyzed during this study are available from the corresponding author upon reasonable request.

## Abbreviations

TSF: Tibial shaft fractures
PMF: Posterior malleolar fracture
CT: Computerized tomography
MRI: Magnetic resonance imaging
PRISMA: Preferred Reporting Items for Systematic Reviews and Meta-Analyses
RCT: Randomized controlled trial
