# Supplementary material for: Incidence and missed diagnosis risk of occult posterior malleolar fractures associated with the tibial shaft fractures: a systematic review
Source: J Orthop Surg Res. 2021 Jun 1;16:355. doi: 10.1186/s13018-021-02502-6 (PMC8167951; doi:10.1186/s13018-021-02502-6)
Supplement: Supplementary file 2 — Additional file 2. Methodological Quality Assessment. [file 13018_2021_2502_MOESM2_ESM.docx]

**Table, additional file 2.** Methodological Quality Assessment

| **Paper** | **Year** | **Subject** | Was the objective of the study clearly defined? | Were includion criteria described? | Were excludion criteria described? | Was a patient satisfaction or outcom score reported? | Was the surgical technique reported adequately? | Was the length of follow-up reported? | **Quality Assessment Grade^a^** |
| --- | --- | --- | --- | --- | --- | --- | --- | --- | --- |
| **Böstman [1]** | 1988 | TSF/spiral TSFs | Yes | Yes | No | Yes | Yes | Yes | High |
| **Georgiadis et al. [11]** | 1996 | TSF | Yes | Yes | Yes | Yes | Yes | No | High |
| **Kukkonen et al. [20]** | 2006 | TSF | Yes | No | No | No | Somewhat | No | Low |
| **Stuermer et al. [18]** | 2008 | TSF | Yes | Yes | No | Yes | Yes | Yes | High |
| **Schottel et al. [14]** | 2014 | TSF | Yes | Yes | Yes | Yes | Yes | No | High |
| **Tsai et al. [21]** | 2014 | TSF | Yes | Yes | Yes | Somewhat | Yes | Yes | High |
| **Jung et al. [23]** | 2015 | TSF | Yes | Yes | Yes | No | Somewhat | No | Moderate |
| **Kempegowda et al. [22]** | 2016 | TSF | Yes | Yes | Yes | Yes | Yes | No | High |
| **Zhang et al. [24]** | 2018 | TSF | Yes | Yes | Yes | Somewhat | Yes | No | Moderate |
| **Huang et al. [6]** | 2018 | TSF/spiral TSFs | Yes | Yes | Yes | Yes | Somewhat | No | Moderate |
| **Hendrickx et al. [25]** | 2019 | TSF | Yes | Yes | No | Yes | No | No | Moderate |
| **Hendrickx et al. [8]** | 2019 | TSF/spiral TSFs/Distal 1/3 TSFs | Yes | Yes | No | No | No | No | Low |
| **Hou et al. [2]** | 2009 | spiral TSFs | Yes | Yes | No | Somewhat | Yes | No | Moderate |
| **Purnell et al. [3]** | 2011 | spiral TSFs/Distal 1/3 TSFs | Yes | Yes | Yes | Yes | Yes | No | High |
| **Warner et al. [4]** | 2014 | spiral TSFs | Yes | Yes | Yes | Yes | Somewhat | No | Moderate |
| **Chen et al. [5]** | 2018 | Spiral TSFs | Yes | Yes | Yes | Somewhat | Yes | Yes | High |
| **Sobol et al. [7]** | 2018 | Spiral TSFs/Distal 1/3 TSFs | Yes | Yes | Yes | Somewhat | Yes | Yes | High |
| **Mitchell et al. [9]** | 2019 | spiral TSF | Yes | Yes | No | Yes | Yes | Yes | High |
| **van der Werken et al. [26]** | 1988 | Distal 1/3 TSFs | Yes | Yes | No | Yes | Somewhat | No | Moderate |
| **Boraiah et al. [13]** | 2008 | Distal 1/3 TSFs | Yes | Yes | No | Somewhat | No | No | Low |
| **Boutin et al. [27]** | 2017 | Distal 1/3 TSFs | Yes | Yes | Yes | Yes | No | No | Moderate |
| TSF, Tibial shaft fracture; ^a^ Grading Criterion: High = 5-6 “Yes”; Moderate = 3-4 “Yes”; Low = 1-2 “Yes” | | | | | | | | | |
