# Supplementary material for: Incidence and missed diagnosis risk of occult posterior malleolar fractures associated with the tibial shaft fractures: a systematic review
Source: J Orthop Surg Res. 2021 Jun 1;16:355. doi: 10.1186/s13018-021-02502-6 (PMC8167951; doi:10.1186/s13018-021-02502-6)
Supplement: Supplementary file 3 — Additional file 3. Results of risk of bias assessment of the individual studies with scores per item. [file 13018_2021_2502_MOESM3_ESM.docx]

**Table, additional file 3** . **Results of risk of bias assessment of the individual studies with scores per item**.

| **Paper** | **Random sequence generation** | **Allocation concealment** | **Blinding of participants, personnel, and outcome assessors** | **Incomplete outcome data** | **Selective outcome reporting** | **Other sources of bias** | **Summary** |
| --- | --- | --- | --- | --- | --- | --- | --- |
| **Böstman [1]** | high risk | high risk | high risk | high risk | high risk | low risk | 1 |
| **Georgiadis et al. [11]** | high risk | high risk | high risk | high risk | low risk | low risk | 2 |
| **Kukkonen et al. [20]** | high risk | high risk | high risk | low risk | low risk | low risk | 3 |
| **Stuermer et al. [18]** | high risk | high risk | high risk | low risk | low risk | low risk | 3 |
| **Schottel et al. [14]** | high risk | high risk | high risk | high risk | high risk | low risk | 1 |
| **Tsai et al. [21]** | high risk | high risk | high risk | low risk | low risk | low risk | 3 |
| **Jung et al. [23]** | high risk | high risk | high risk | low risk | low risk | low risk | 3 |
| **Kempegowda et al. [22]** | high risk | high risk | high risk | low risk | low risk | low risk | 3 |
| **Zhang et al. [24]** | high risk | high risk | high risk | low risk | low risk | low risk | 3 |
| **Huang et al. [6]** | high risk | high risk | high risk | low risk | low risk | low risk | 3 |
| **Hendrickx et al. [25]** | high risk | high risk | high risk | low risk | low risk | low risk | 3 |
| **Hendrickx et al. [8]** | high risk | high risk | high risk | low risk | low risk | low risk | 3 |
| **Hou et al. [2]** | high risk | high risk | high risk | low risk | low risk | low risk | 3 |
| **Purnell et al. [3]** | high risk | high risk | high risk | low risk | low risk | low risk | 3 |
| **Warner et al. [4]** | high risk | high risk | high risk | low risk | low risk | low risk | 3 |
| **Chen et al. [5]** | high risk | high risk | high risk | low risk | low risk | low risk | 3 |
| **Sobol et al. [7]** | high risk | high risk | high risk | low risk | low risk | low risk | 3 |
| **Mitchell et al. [9]** | high risk | high risk | high risk | low risk | low risk | low risk | 3 |
| **van der Werken et al. [26]** | high risk | high risk | high risk | low risk | low risk | low risk | 3 |
| **Boraiah et al. [13]** | high risk | high risk | high risk | low risk | low risk | low risk | 3 |
| **Boutin et al. [27]** | high risk | high risk | high risk | high risk | high risk | low risk | 1 |
